# How digital technologies reshape marketing: evidence from a qualitative investigation

**DOI:** 10.1007/s43039-023-00063-6

**Published:** 2023-01-17

**Authors:** Federica Pascucci, Elisabetta Savelli, Giacomo Gistri

**Affiliations:** 1 Department of Management, Polytechnic University of Marche, Ancona, Italy; 2 Department of Economics, Society, Politics, University of Urbino Carlo Bo, Urbino, (PU) Italy; 3 Department of Political Science, Communication and International Relations, University of Macerata, Macerata, Italy

**Keywords:** Marketing, Digital transformation, Customer engagement, Market analytics, Marketing skills, Marketing organisation

## Abstract

Digital technologies are now imperative for markets and society, and digital transformation is becoming a key area of business innovation. However, digital transformation is complex, and firms still lack the abilities to fully grasp and exploit its opportunities. This study investigates how digital technologies are currently implemented by companies. In particular, since digital transformation can reshape the traditional process of value creation in which marketing is primarily involved, the article analyses the impact of digital transformation on traditional marketing, including its role, organisation, and instruments. The study conducted qualitative research in the form of in-depth interviews with managers working for companies operating in different Italian industries. The results show that digital technologies are widely used by firms, although they often belong to the category of traditional tools, and companies are more ‘digitalised’ than ‘digitally transformed’. Digital technologies impact marketing by improving the abilities of market analytics, pricing, and channel management and helping to build relationships with clients to achieve value co-creation. Professional skills are variously augmented, while organisational processes are becoming more effective and flexible through the use of multiple knowledge and cross-functional experiences. Research and managerial implications are discussed in light of the main barriers and risks involved in the implementation of digital transformation.

## Introduction

Society is undergoing a constant technological transformation that involves changes in all areas – from the economy to culture and politics. The business world is profoundly influenced by this transformation, which is occurring across industries, albeit at varying intensities. Data from the most recent report by Anitec-Assinform (2021) indicate that TLC & media, industry, and banking are the three sectors in Italy that reveal the highest investments in absolute value totalling €8.815, €7.909, and €7.989 million, respectively. In this context, some technologies can be considered already mature, such as mobile business, cloud computing, and the Internet of Things (IoT), whereas other technologies seem to be emergent, including blockchain and artificial intelligence. In the industrial sector, the digital investment mainly aims to improve customer engagement, enhance relationships with employees, reduce time-to-market through agile manufacturing and supply chain management, promote operational efficiency, and advance data exploitation.

In light of this evolution, there is growing interest in the topic of digital transformation (DT). Both academics and practitioners have proposed several definitions of DT, which has thus become a ‘hype’ or ‘buzzword’; however, not enough attention has been paid to what DT is and how it can be conceptualised (Gong & Ribiere, 2021; Hausberg et al., 2019). Scholars agreed that the implications of DT are profound and manifold. Moreover, DT extends beyond the adoption of one or more digital technologies, as it entails rethinking the market approach and value proposition, changing organisational processes and structures, and, in some cases, innovating the business model, thus involving a plurality of internal and external actors (Broekhuizen et al., 2021).

In this context, marketing can play a fundamental role, considering its important function of connecting firms and the market. It can help a company adapt to the constantly changing needs and trends coming from the external environment. However, to address the challenges of DT, marketing theory has to be enriched with new concepts, logic, and tools that are in line with the ongoing evolution (De Luca et al., 2020). According to Kumar et al., (2021), the future of marketing lies in firms’ efforts to acquire a holistic understanding of their customers’ needs and behaviours across platforms, devices, and varied products and services. To this end, it is fundamental to investigate the impact of new digital technologies on marketing strategies both to understand how they are currently being leveraged and to identify potential areas that merit deeper exploration.

To answer these calls, the present study explores how DT is currently perceived and implemented by companies and how it affects marketing by influencing its role, organisation, and internal processes. The main objective is to understand the complex relationship between DT and marketing by investigating (i) how DT can be leveraged by marketing processes and resources; and (ii) how DT can improve the value creation process in which the marketing function is involved. Existing literature on this topic is quite fragmented, as studies have considered one single marketing activity or decision, and the findings have often been discordant. Thus, further analyses are recommended.

Specifically, the contribution of this exploratory analysis is twofold. First, unlike existing studies, the present research considers marketing as a whole strategy and process, thus generating a holistic view of DT implications. In this respect, the findings reveal some important changes concerning the role and organisation of marketing and the management of its activities. Second, the study identifies potential risks and barriers to DT encountered by the firms and how companies are trying to overcome them. From these risks and barriers, we derive some useful recommendations for managers and policymakers.

The paper is structured as follows. Following this introduction, Sect. 2 describes the theoretical background of the study and establishes four research questions. Section 3 sets the methodology, and Sect. 4 presents the findings of the multiple interviews. Section 5 discusses the implications for theory and practice, and Sect. 6 concludes the paper by highlighting both the limitations of the study and future research directions.

## Theoretical background

### Digital transformation

The term ‘digital transformation’ has entered the agendas and debates of both scholars and practitioners, and the concept is currently a ‘hot topic’. Conceptual and empirical research on DT has grown enormously in recent years in different fields, and many literature reviews have been published (Krishen et al., 2021). In the Scopus database, the number of articles, conference papers, books, and book chapters containing ‘digital transformation’ in the title, keywords, and abstract increased from 2 to 3,109 between 2000 and 2021.

The topic of DT can be investigated from two key perspectives: that of the firm which adopts digital technologies or that of the customers at whom the company’s actions are directed. This paper focuses on the first perspective, which recognises the multidisciplinary nature of the phenomenon in involving changes in strategy, organisation, technologies, supply chains, and marketing (Verhoef et al., 2021). For companies, digital technologies may enhance operational efficiency and the effectiveness of existing processes (Pascucci, 2017). Moreover, they can provide new opportunities to enable business model innovations (Ancillai et al., 2022), such as digital servitisation (Frank et al., 2019; Gebauer et al., 2021; Grandinetti et al., 2020).

According to this dual nature of DT implications, it is possible to distinguish three stages of DT (Verhoef et al., 2021): digitisation, which is the encoding of analog information into a digital format; digitalisation, which is the adoption of digital technologies to improve existing business processes; and DT, which is the development of a new business model based on digital technologies. In line with this conceptualisation, DT is the most pervasive and complex stage of transformation (Gong & Ribiere, 2021), and most firms, especially SMEs, are still in the second stage. Each stage places specific demands on firms’ digital resources, organisational structure, growth strategy, and metrics. As a result, DT can have different levels of maturity as a changing process and there is not ‘one best way’ to implement DT; rather, each firm needs to devise its approach (Checchinato et al., 2021).

Although the growing number of studies on DT, some authors have argued that the phenomenon remains under-investigated (Fernandez-Ravira et al., 2021), and there is still a need to understand the true nature of change and transformation (Gong & Riviere, 2021). Moreover, despite the opportunities presented by technological progress, it seems that firms are not yet fully exploiting the potential of digital technologies, as many companies have failed to obtain the expected results. Thus, DT represents ‘a substantial problem of practice’ (Hinterhuber et al., 2021, p. 3). In this regard, Gebauer et al., (2020) have highlighted a ‘digitalisation paradox’ in which ‘companies invest in digitalisation but struggle to earn the expected revenue growth’ (p. 315). In light of this, our first research question (RQ1) is as follows:

#### RQ1

How do firms conceive of DT? In particular, how and why do they leverage digital technologies for it?

### Marketing in the age of DT

Marketing is one business area witnessing DT on an intense scale and experimenting with new technologies, such as artificial intelligence, blockchain, and the IoT (Grewal et al., 2020; Kumar et al., 2021). Digitalisation has radically transformed the customer journey since individuals are now constantly connected. Moreover, the era of digital technologies features exceptional growth in customer empowerment. Because of the abundance of information and interaction opportunities, consumers no longer accept the role of passive recipients of firms’ messages (Auh et al., 2019; Akhavannasab et al., 2018). This change requires innovative approaches to marketing communication and forces brands to interact with individual customers quickly, openly, and continuously.

All of these trends have led companies to adopt a customer-centric approach, which prioritises the customer first and foremost in their organisational strategies (Shah et al., 2006). Consequently, relationships between firms and their customers are evolving, and firms should invest in building stronger, closer, and longer-lasting customer relationships in both B2B and B2C markets. Digital technologies have helped firms in pursuing this aim. For instance, with CRM technologies, companies can gather customer information through many touchpoints and share the needed information across the company to align marketing decisions with customers’ needs and values, identify more profitable customers, guide investment decisions, quickly respond to customer requests, and deliver customised offerings and experiences (Nasir, 2015).

Customer data form the basis of a customer-centric organisation. Data are assuming an increasingly central role in marketing as a fundamental resource for building and maintaining customer relationships, personalising products, services, and the marketing mix, and automating marketing processes in real-time. Nowadays, firms operate in ‘data-rich environments’ (Wedel & Kannan, 2016), such as web and social media, which have brought an explosion of real-time data, especially unstructured ones, that can reshape the management of marketing activities and thereby afford new business opportunities.

The combination of data proliferation, algorithmic advancement (artificial intelligence), and more powerful computing and storage capabilities supports the transformation of data into business insights, decisions, and actions, thus allowing for the development of a marketing ‘data-driven approach’ (Anderson, 2008; Pascucci et al., 2022), which helps companies to customise products and optimise marketing decisions for customers on a real-time basis (Wirtz et al., 2017).

Because of the growing relevance of data-driven decisions, marketing analytics has become central to modern organisations and now represents a new frontier in marketing research (Sheth, 2021). However, the increased volume and variety of data remain mostly untapped by firms, and many organisations have failed to incorporate the data effectively in their decision-making processes (Tabesh et al., 2019). Thus, many firms have not effectively realised their ‘big data dreams’ (Mazzei & Noble, 2017). Previous research has identified a shortage of organisational resources (e.g. infrastructure, human resources, and competencies) and cultural and technological barriers (Tabesh et al., 2019) as the main reasons for this failure. Organisations that aim to profit from (big) data analytics, indeed, should have the following elements: first, a culture and leaders that recognise the importance of data, analytics, and data-driven decision making; second, a governance structure that prevents silos and facilitates the integration of data and analytics into the organisation’s overall strategy and processes; and third, a critical mass of marketing analysts who collectively provide sufficiently deep expertise in analytics as well as substantive marketing knowledge (Wedel & Kannan, 2016).

Anyway, this evolution is transforming the way marketing strategies are developed and implemented, and produces an overall reshaping of the marketing mix management (Caliskan et al., 2021). As a result, a new marketing approach (i.e., Digital Marketing: Krishen et al., 2021), organisation, and skills are required. Notably, scholars have developed the new ‘Marketing 4.0’ (Kartajaya et al., 2016; Jara et al., 2012), which is based on the assumption that customers are not only looking for products and services to satisfy their needs but also demanding to be part of the production process. This aspect calls for a shift towards a more collaborative, interactive, and inclusive approach, which Marketing 4.0 tries to enable to improve brand and customer relationships (Dash et al., 2021).

For marketing policies, the rise of social media has dramatically changed how firms manage their brands. Consumers have become ‘pivotal authors’ of brand stories, and firms need to pay attention to such consumer-generated content and coordinate firm-generated content accordingly (Gensler et al., 2013).

The participatory role of customers is also at play in the product customisation enabled by digital technologies, such as virtual, augmented, or mixed reality, that have the potential to not only increase customer participation in the new product design (Mourtzis & Doukas, 2012) – but also to create new types of improved customer experiences in all steps of the customer journey (Hoyer et al., 2020; Flaviàn et al., 2019). Notably, heterogeneity exists in consumer demand for customisation, and a better understanding of these differences is vital for brand managers to effectively develop and deliver customisation opportunities for consumers (Pallant et al., 2020).

Digital transformation also impacts pricing decisions. The growing availability of data and pricing algorithms is enabling personalised and dynamic pricing, in which prices can change by the day, every hour, or for each customer according to the data that are collected and analysed. This widespread practice is especially prevalent in the service sector (Abrate et al., 2012).

Finally, DT allows consumers to utilise and move seamlessly across multiple channels in their customer journey (Hansen & Sia, 2015). Along with these developments, ‘omnichannel management’ is increasingly emerging as a new strategic approach – in contrast to ‘multichannel management’ – to manage the simultaneous and synergetic interplay between channels and thus provide the seamless customer experience that customers expect (Verhoef et al., 2015).

In the wake of all of these evolution trends, digital marketing studies have increased substantially over the past 20 years. Nevertheless, digital marketing is still growing and has not yet reached maturity (Krishen et al., 2021). In addition, the existing literature is quite fragmented as previous research is mainly focused on specific marketing activities, and findings have often been discordant. Therefore, our second research question (RQ2) is as follows:

#### RQ2

what are the impacts that DT is currently having on marketing activities and firm-customer relationships?

While digital changes present numerous opportunities, they undoubtedly pose some challenges as well. Leeflang et al., (2014) have already identified three main challenges for digital marketers: the ability to generate and leverage deep customer insights, the management of brand reputation in a marketing environment where user-generated content is predominant, and the assessment of the effectiveness of digital marketing. The issue of human resources and capabilities remains a particularly prominent topic, as there is a widening gap between the accelerating complexity of markets and technologies and the evolution of firms’ digital marketing capabilities. Increased data complexity creates a ‘digital talent gap’ (Leeflang et al., 2014) that is aggravated by the difficulty of finding employees who are capable of combining quantitative and analytics skills with marketing skills. In this regard, Herhausen et al. (2021) have identified two marketing capability gaps: the ‘practice gap’, concerning the deficit between managers’ current practices and their ideal digital marketing capabilities; and the ‘knowledge gap’, which is the divide between the digital marketing transformation and the extant scholarly knowledge that underpins it. Matarazzo et al., (2021) have found that dynamic capabilities, especially sensing and learning capabilities, are generally fundamental facilitators of DT in SMEs. According to these authors, DT is a learning process, as it requires new human resources and some changes to organisational structure, which demonstrates the relevance of the ‘soft’ dimension of DT as opposed to the technological dimension. Likewise, Eller et al., (2020) have determined that the recruitment of employees with the required skills is a significant barrier to successful digitalisation among SMEs, and a digital strategy with concrete key performance indicators and actions to monitor the process is a fundamental driver of digitalisation. In particular, there is a need to more closely align marketing and information technology (IT) in terms of knowledge and capabilities (Graesch et al., 2021).

Authors have largely agreed that scarce attention has been given to the related competencies that firms need to fully exploit the potential of digital technologies. It could be interesting and useful to investigate the reason for this gap, study how digital technologies are being leveraged, and identify the most critical areas. Thus, our third research question (RQ3) is as follows:

#### RQ3

What is impact of DT on marketing organization and competences?

A further debate in the marketing literature concerns the implications of DT for the role and relevance of marketing within the company. According to some scholars, marketing is losing its influence and has become marginalised, as marketing decisions have moved to other departments (Homburg et al., 2015; Verhoef & Leeflang, 2009). In the same vein, Quinn et al., (2016) have stated that evolution ‘has precipitated a managerial sense of crisis for marketing, triggering a transformation that has repercussions for the future of the discipline and its practice’ (p. 2104). In contrast, other authors have posited that technological evolution has contributed over time to the role and scope of marketing within organisations expanding from primarily involving the development and management of creative communication to now including the implementation of data-driven and technology-enabled marketing practices that are not only relevant to firms and customers but also financially accountable (Shah & Murthi, 2021). Based on this discussion, it is interesting to explore how DT may have changed the function of marketing and the main implications of this evolution. Therefore, our fourth research question (RQ4) is as follows:

#### RQ4

How might DT have changed the overall role and importance of marketing within firms?

## Methodology

This research employed a multiple case study methodology to perform an exploratory analysis of DT in 11 Italian firms operating in different industries. Considering the novelty and complexity of DT, the case study approach is particularly suitable, as it emphasises the richness of the phenomenon and deeply grounds the findings in the varied empirical evidence that is collected (Eisenhardt, 1989). Case studies are particularly useful for providing in-depth answers to ‘how’ and ‘why’ research questions, thus supporting a holistic, comprehensive, and realistic understanding of a certain phenomenon. Moreover, Hausberg et al., (2019) have directly called for more case studies that describe the benefits, values, and weaknesses of DT implementations.

The research team conducted in-depth and semi-structured interviews (n = 20) based on face-to-face and online meetings with individuals who had key-roles in DT, including those of chief officer (CO), chief digital officer (CDO), or chief marketing officer (CMO). Semi-structured interviews suited the study’s explorative aim because they allow researchers to follow a structured approach while also leaving space for interviewees to freely talk about their experiences and opinions (Yin, 2009). Each interview lasted nearly two hours, was conducted in Italian, and was audio-recorded, transcribed, and analysed. The number of interviews, within each company, has been determined in line with the principle of theoretical saturation, thus interviews have been conducted till the information gathered has been considered sufficient for the scope of the analysis and no further relevant information could have been added by additional interviews (Strauss & Corbin, 1990).

The interview guide was aimed at exploring DT in terms of four aspects: first, the implemented technologies (technological macrotrends); second, the implications of technology adoption for marketing processes; third, the implications of technology adoption for human resources and organisation; and fourth, the implications of technology adoption for customer relationships. The interview guide was carefully designed based on the previously analysed literature on DT and marketing.

In addition to the primary data from the interviews, secondary data were gathered from newspaper and magazine articles, corporate presentations, companies’ reports, and their own websites. These allowed for the triangulation of data, which provides greater depth to the study of a phenomenon from different perspectives (Denzin, 1984).

**Fig. 1:**
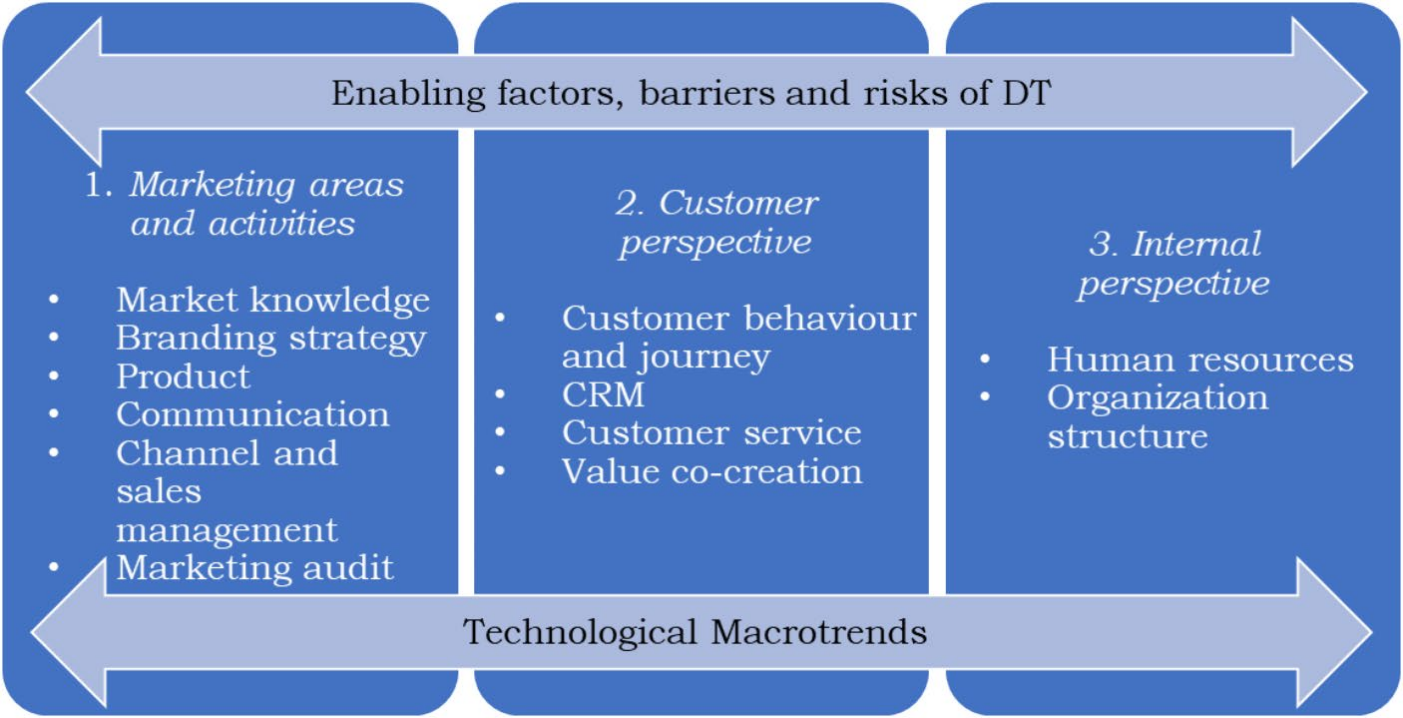
Framework of analysis

Figure 1 depicts the overall framework that guided the case study development and analysis.

As for the sample, a purposeful procedure (Patton, 2005) was used to select firms from the Marche region. This area has n.145.609 firms (2021), which represents the 2.8% of all Italian firms. Manufacturing and commerce are the main sectors. Marche’s entrepreneurial density (i.e., number of firms per inhabitant) is higher than the Italian one (97 firms per 1000 inhabitants vs. 87, https://statistica.regione.marche.it). Such entrepreneurial vocation makes Marche Region an interesting context to investigate the phenomenon of firms’ digital transformation.

Companies have been selected to represent the principal industries of this area and to provide a clear picture of the current situation while considering different sectors, as well as different needs and criticalities from the perspective of the DT phenomenon. Thus, because the intention was to get a broad variety of DT cases, the sample covers various industries and sizes (see Table 1). Moreover, we selected only incumbent firms, as their legacy ensures that they have experience with DT challenges and barriers and must navigate conflicts and trade-offs between existing and new ways of conducting business (Verhoef et al., 2021). Finally, considering our research objective, all the selected firms must be involved in some form of DT, being aware of the importance to invest in new technologies.

Although the analysis has been conducted at a regional level, the findings are significant as Marche is one of the most industrialised regions in Italy, and the distribution of its industries is quite similar to the Italian one, with a prelevance of small and medium sized firms.

**Table 1:** Details of investigated firms

Firms	Industry	2020 turnover (in €)	# of employees	Market
Algam EKO Srl	Musical instruments	19.3 million	55	B2B – B2C
Ariston Thermo Spa	Thermal comfort, burners, and components sectors	1.99 billion	7,743	B2B – B2C
Biesse Spa	Mechatronics	700 million	4,200	B2B
Diasen Srl	Construction	7 million	30	B2B – B2C
Doucal’s Srl	Footwear	12 million	70	B2B
Eden Viaggi - Alpitour Spa	Tour operating	725 million	4,000	B2B – B2C
Go World	Tour operating	2 million	57	B2B – B2C
IT Consult	Consultancy (knowledge management)	1.3 million	18	B2B
Magazzini Gabrielli Spa	Retail	900 million	2,000	B2C
Salumificio Ciriaci Srl	Food	13 million	40	B2B – B2C
Simonelli Group Spa	Mechanical	75 million	200	B2B – B2C

The collected data have been examined following Eisenhardt’s (1989) guidelines for within-case and cross-case analyses. Each case was thoroughly studied to obtain insights into the relationship between DT and marketing. Then, the cases were compared to analyse similarities and differences and gain a richer understanding of the DT phenomenon.

## Results

### Intra-case analysis

DT is an ongoing process in the analysed companies that was variously perceived and implemented. Table 2 shortly depicts the current status of DT within each company, by showing the main technologies adopted and relative business areas of application.

**Table 2:** The current status of companies’ DT

Firms	Digital technologies	Areas of application
Algam EKO Srl	Mobile technology/smart apps Social media	Communication
Ariston Thermo Spa	AI Blockchain Social media	Service Assistance (CRM) Internal Organization Communication
Biesse Spa	Big Data IoT Social media	Branding Service production Communication
Diasen Srl	Social media	Communication CRM
Doucal’s Srl	ERP Social media Smart glasses	Production Communication Training
Eden Viaggi - Alpitour Spa	Big data Blockchain Mobile technology/smart apps Social media	Service production Communication
Go World	Big data Social media	E-commerce Communication
IT Consult	Josh platform MailChimp Microsoft Dynamics Social media	Production Email automation CRM Communication
Magazzini Gabrielli Spa	Big data ERP Mobile technology/smart apps Social media	Service production Promotion Communication
Salumificio Ciriaci Srl	Mobile technology/smart apps Social media	Production Communication
Simonelli Group Spa	AI Big Data IoT Mobile technology/smart apps Social media	Service production Product maintenance Communication

Algam EKO operates in the musical instruments industry. The company employs digital technologies mostly to interact with customers and suppliers. Specifically, it has been using mobile technology/smart apps since 2016 and is active on social media since 2009, especially on YouTube, Facebook, LinkedIn, and Instagram. AI applications are in progress, intending to bring online the experience that is lived in physical retail, by creating ad hoc videos in collaboration with retailers. Algam EKO doesn’t use IoT or Blockchain technologies yet.

Ariston Thermo works in the thermal comfort, burners, and components sectors. Since it is a multinational company that grew up through many acquisitions, digital technologies have been used to create greater harmonization within the group. They started with assistance services. Having sensors inside the boilers the company would be able to know if there is a malfunction and, therefore, intervene earlier, sometimes solving the problem completely remotely without going to customers’ homes. This brought a very different customer service model and also important business insights with data collection, storage, and utilization. About AI and IoT the company is at its beginning. The integration between IoT and Blockchain remains an objective of the near future. Also for digital communications, Ariston Thermo is in its infancy. It is present on social media platforms but it needs to find its soul, as it is still very jagged because social media are not managed centrally and strategically. As the Chief Digital Officer said:

*Everyone does his own so we are still at the stage of trying to understand who is doing and what to bring it back to a trend*. (Ariston Thermo Spa)

Biesse operates in the mechatronics industry. The company launched “Sophia” in 2016, an Industrial Internet of things (IIoT) platform based on sensors connected to machinery, which monitor the activities of customers and generate real-time information. Sophia allows cloud computing for data management in open systems; big data analytics for the optimization of products and services as well as production processes; IIoT as multidirectional communication between products and customers’ processes. The advent of new digital technologies had a positive impact also on the branding strategy of the Group, involving a greater presence of the brand in digital channels such as social media platforms, which contributed to redefining the brand as more innovative, digital, clear, and simple.

Diansen owns the construction industry. Recently, an increasing number of customers have begun to adopt social media, especially Instagram, to find specific content once asked by professionals. Customers have become increasingly active and started to interact with the company through these channels so, since 2017, the company has decided to increase its presence on social media, by enriching the content offer, the level of interaction, and the users’ engagement. DT pushed the company to be more effective in digital marketing by using tools such as email marketing platforms, digital content platforms, and CRM software. Diansen didn’t start using more sophisticated applications such as Big Data, AI, or Blockchain yet, even if they consider AI potentially useful for production processes.

Doucal’s works in the footwear industry. Digitization was driven primarily by the evolution of the external context. In Doucal’s, DT started from production and then came to marketing, as a result of the original company’s orientation on the product. Marketing has always meant communication and, recently, social media communication (since 2015). Doucal’s did not introduce AI, Blockchain systems, IoT, or mobile technologies yet but it has been using Enterprise Resource Planning (ERP) software since 2018 to automate and manage core business processes. Considering production, artisans involved in cutting, wear smart glasses with a micro camera to capture the actions in real-time. Filming is carried out on anonymous workstations and divided according to both the shoe model and the specific processing. In this way, the intangible assets become explicit and usable even at a distance and on different platforms for example for the training of new personnel.

Eden Viaggi – Alpitour is a tour operator. In July 2016, it has been released the app “My Alpitour World” to be the personal travel assistant of customers at every stage of their journey, from booking to their return, to offering personalized services during their holidays. To find and interact with customers Eden Viaggi – Alpitour mostly used social media, since 2010. In particular Facebook, Instagram, and YouTube. So far, the company looked at AI with skepticism in its industry, especially considering the use of chatbots to interact with customers and the marketing director remarked that it is difficult to have questions that can be so standardized as to have an adequate answer from a machine or an algorithm. Since 2019, the company is also using Big Data and analytics to develop customized forecasts and commercial proposals. While IoT technologies did not have been implemented, they are experiencing Blockchain and an NFT is being developed within the hotel division.

Go World is another tour operator. The company considers AI, IoT, and Blockchain technologies more suitable for larger players in the sector where decision-making and control processes are more complex. In the same way, Go World did not implement mobile technology/smart apps because they think are more appropriate in B2C contexts than in B2B, where most of the company business is located. On the other hand, the company uses social media to interact with its customers and in 2019 it started to plan a new e-commerce platform that uses Big data and analytics to commercialize customized products. Besides, an e-learning platform has been used for travel agent training.

IT Consult works in the knowledge management industry. Specifically, it develops software for document management using the “Josh platform”. The company is therefore fully involved in the dynamics of digital transformation. The main challenge concerned the definition of digital marketing activities and their integration with other established commercial actions. IT Consult did not practice IoT, Blockchain, or AI but it uses social media to communicate and interact with its customers, mainly YouTube, LinkedIn, and Facebook. The company also habits marketing automation tools in mailing such as “MailChimp” and Microsoft Dynamic for CRM.

Magazzini Gabrielli is a big retailer operating in the large-scale distribution industry. Digitalization involved particularly communication activities, which were usually based on distinct physical and digital channels. Most of the endeavors went to the need for integration following the omnichannel trend. The company also used Big data and analytics to plan marketing actions along the customer journey. To communicate and send promotions, Magazzini Gabrielli started in 2020 using social media platforms, specifically Facebook, Instagram, and LinkedIn. In 2021 it has been launched also the OasiTigre app that operates as a kind of digital fidelity card. The company did not use AI, Blockchain, or IoT technologies yet.

Salumificio Ciriaci operates in the food industry. It was founded in 1937 with the idea of producing quality, healthy, and genuine products processed with traditional methods. Recently, there was a generational and managerial change and it has been launched a rebranding strategy to link the quality of the product to the quality of the territory enhancing the supply chain. In this regard, it has been implemented a communication strategy through social media. In 2020 the company also introduced a cold room monitoring system with an app. However, till now, it didn’t employ IoT, AI, or Blockchain technologies.

Finally, Simonelli Group owns the mechanical industry. The company doesn’t have used Blockchain technology yet. There is an ongoing project about IoT to support product preventive maintenance. They are also experiencing Big Data applied to CRM. The company uses social media and started to develop an app system related to products. It is a system made up of a connected machine and two platforms: the former is more technical while the latter is more oriented to the coffee culture that supports customers and service connoisseurs. So DT has helped in offering a system package to the customers, rather than a chain between distributors and customers.

### Cross-cases analysis

#### How DT has been conceived and performed in business practice

The above analysis depicts a very fragmented scenario, characterised by the adoption of several technologies by the analysed companies, according to different aims and business purposes.

Overall, social media emerged as the most widely used digital technology that companies employ to both communicate and interact with customers, based on a different product or channel page. Despite the social media presence has been usually established for less than five years, the growing investment in such technologies reflects an increasing interest in their current and future adoption. Moderate interest was found in mobile technologies and smart applications. As seen before, Salumificio Ciriaci recently launched an application allowing for automated monitoring of the cold rooms, while Go World anticipated the launch of a multimedia application catalog in 2022 based on high-resolution photographs describing tourism offerings to the final customers. More advanced technologies, such as artificial intelligence applied to production or preventive maintenance, Big Data processing software, blockchain systems, and IoT applications were still under-employed, especially among the smallest firms. Notably, only some companies in the mechanical sector (i.e. Biesse and Simonelli Group) used digital automation and simulation software between machines to improve the overall efficiency of production processes. On the other hand, some respondents highlighted the use of ERP software connected to CRM strategies and tools applied to both customers and sales management (i.e., Doucal’s Srl; Magazzini Gabrielli Spa).

The heterogeneous (and sometimes sparse) adoption of digital technologies by companies is due to several factors, which have been recognised as potential facilitators of or barriers to the digitalisation process. The main facilitators emerging from the interviews can be grouped according to their internal or external origin. Meanwhile, the barriers can be categorised into cultural factors, change management obstacles, or perceived risks associated with DT. Table 3 provides a brief description of such variables.

**Table 3:** Digital transformation facilitators and barriers

	Internal factors		External factors	
Facilitators	Management awareness of DT benefits in terms of efficiency, speed, re-skilling opportunities, and market/demand monitoring		Trends in consumer behaviour, which appears increasingly dynamic, digital, interactive, and demanding continuous assistance	
	Management willingness to improve market visibility through the use of digital technologies for developing targeted, less expensive, and more impactful communication		Thrusts of competitors and digital advancement of the main players	
	Management involvement in DT processes encourages an active and top-down approach to DT		Reduction of economic barriers and increased government incentives (i.e. National Industry Plan 4.0/2017), which make digital technologies more accessible for all firms regardless of the availability of their financial resources	
	Recognition of digital services as a way to improve the company’s ability to provide quick customer response and increase market monitoring		Labour market trends are characterised by the increasing availability of new skills linked to the use of digital technologies	
	Trust-based relationships with external providers can assist companies in their digital challenges		The COVID-19 pandemic has been recognised as a ‘potential accelerator’ of the digitisation process by forcing new consumption and purchasing habits and necessitating the modification of traditional firm/customer relationship management	
Barriers	*Cultural factors*	*Change management obstacles*		*Perceived risks*
	In-depth cultural changes are required, especially among ‘analog born’ who are not familiar with digital technologies based on immediacy and data sharing	Digital transformation requires new skills and organisational changes		Digital transformation can imply the risk of control-losing, which occurs when companies exclusively focus on the technological transition without considering the cultural and organisational changes it requires
		Digital transformation requires a proper managerial approach, which can be top-down or bottom-up by nature, according to the specific characteristics of the company		Digital transformation requires continuous investments by firms to face technological obsolescence, which could be difficult to support long term
				The DT process of a company could be misaligned with that of their customers and/or suppliers, thus weakening the stability of market relationships

The above factors influenced the way companies invested in digital technologies and implemented digital tools within the organisation as well as the different impacts of DT on firms’ activities and processes, especially the marketing function.

#### The role of DT in supporting customer/firms relationships

The increasing use of digital technologies radically changed customer behavior and journey.

The investigated companies recognized that the COVID-19 pandemic has led to a growing willingness on the part of the demand to use digital means to inform themselves, learn about and make purchases, and communicate in both B2B and B2C contexts. Customers have become particularly focused on the digital presence of companies with which they wish to engage, with particular attention to websites and social media pages:

*Today, the first point of contact is online, and if we miss the opportunity to make a good impression on the buyer, we risk losing his interest. It is useless to have the best product in the world if you do not know how to present yourself.* (Salumificio Ciriaci Srl)

Some companies (e.g., Algam EKO Srl, Ariston Thermo Spa, and Biesse Spa) noted how the digital revolution has led to a sort of ‘hybridisation’ between the customer journey of B2B and B2C customers:

*Compared to the past, we have to completely rethink the way we interact with our customers. Today, our customers are looking for new machines online in the same way a consumer is looking for a new smartphone. We are still biased towards B2B logic, but we will have to evolve towards B2B models with B2C logic.* (Biesse Spa)

Similarly, Ariston Thermo, while recognizing different types of key-customers (i.e., end-user, installer, technical assistance center, designer, and distributor) considered the importance of DT to define a shared vision among them. Digital technologies, indeed, allowed the company to better interact with the different actors, thus improving its overall customer-centric approach and ability to provide customer-based solutions:

*It is necessary to develop a clearer vision of the final purchase process: ‘which digital outlet is contacted?’ ‘How did the customer come into contact with the company?’ ‘How is it possible to bring him on board?’ ‘How can we keep him engaged?’ ‘How can we measure his satisfaction?’ ‘How can we support him?’ To date, the answers to these questions are almost everywhere in the company, but they have never been seen with a customer-centric approach. Once this reasoning has been finalised, hybrid solutions can be developed for other customers in the B2B and B2C contexts.* (Ariston Thermo Spa)

Hence, digital technologies usually evolved the customer journey into a more dynamic and interactive process, based on multiple touchpoints through which customers can meet companies and their brands, inform themselves about products and easily specify their needs, expectations, experiences, and (dis)satisfaction. As a result, the investigated companies recognised a greater ability – and propensity – of their customers to collaborate in the value creation process, to achieve tailor-made solutions. Doucal’s Srl, for example, noted that customers today are much more focused on data than in the past and are willing to share data with the company:

*We collaborate much more to understand the needs and expectations of the final consumer. Thanks to the amount of data that can be collected online, new prospects for collaboration with the retail world are opening up.* (Doucal’s Srl)

Similarly, Eden Viaggi - Alpitour recognised that interactive communication on social media has enabled customers to learn about a travel experience before undertaking it:

*Customers are increasingly used to moving between channels at different stages of their customer journey. Many begin to inquire online and then complete the purchase at the agency.* (Eden Viaggi - Alpitour Spa)

In this way, customers can increasingly contribute to the co-planning and co-creation of their trip, thus increasing the possibility to achieve more modular packages and make travel changes with greater ease and speed.

The above changes in consumer behavior and the customer journey influenced the firms-customers relationships and how companies manage them. Overall, relationships were intensified as digital technologies allow customers to be reached online, have their technical problems resolved through video-remote assistance, shop online, and enjoy faster and more streamlined interactions with suppliers.

From a technological standpoint, the main tool companies used to manage customer relationships is CRM, which has been recently strengthened to better predict demand, optimise the effectiveness of the sales team, manage contacts, and provide after-sales assistance. Through CRM, companies can benefit from a constantly updated customer history, which they can use as a basis for planning more targeted and timely marketing actions. In many cases, CRM is conceived of as a sort of ‘facilitator’ which was introduced to satisfy the need to track the customer journey more systematically and to interact with the end-user. Furthermore, CRM was used to reduce information asymmetry, simplify the purchasing process by enabling companies to offer customers the most relevant content and products for their needs, and make the sales process more flexible, as the continuous evolution of sales portals allows for selecting or building customized packages and products. Relationships with customers could only benefit from these technological advances.

However, relationship management has also benefited from a different way of managing traditional marketing activities, under the effect of DT. First of all, DT has improved the overall market knowledge and companies’ ability to understand market trends, opportunities, and threats, thus enhancing current and future strategic awareness. Specifically, the increased application of digital tools resulted in significant improvement in customer knowledge thanks to the introduction of specific software, which improved the sales control, as well as the use of social media insights or web analytics enhancing the firms’ ability to address customer expectations and provide targeted marketing proposals:

*We do a lot of analysis related to social network insights; we work a lot on web analytics by connecting them with many other data sources. This allows us to analyse in-depth our current and potential customers as well as the customers of our competitors. We are also experimenting with the use of portals that allow complex data analysis, such as Hubspot.* (Simonelli Group Srl)

The management of marketing policies has been adapted consequently.

In terms of product, the increasing market knowledge and the possibility to interact with the demand, combined with the adoption of specific software and digital technologies, improved the companies’ ability to provide more innovative and customized solutions. Advanced design software and product lifecycle management platforms have been considered critical tools enabling product simulations and better time-to-market performance (e.g., Ariston Thermo Spa). In some cases, the adoption of virtual reality applications (e.g., Ariston Thermo Spa, Biesse Spa) reduced the need for field tests, resulting in cost savings and shorter lead times. Besides innovation, companies recognized that DT enhances product value enrichment by means of digital sensors that make the product easily connected and more functional. Even in cases where digitalisation mainly concerned service components, companies perceived an increase in their overall value offering due to enrichment of the product’s intangible components. In this regard, the Biesse experience has been emblematic, trough the launch of the ‘Sophia project’, which allowed to provide a new generation of services, including the monitoring of machines’ production, the preventive maintenance, the improvement of customer assistance for maintenance activities, and the overall reduction of delivery times - which avoid downtime and production interruptions. Even in other companies having a more traditional production concept compared to Biesse Spa, it was explicitly stated that digital applications were introduced to support the service offering. For example, Diasen Srl had recently added a Chatbot to the company website, aimed at delivering real-time support in the early stages of the customer journey. Similarly, Algam Eko had developed ad hoc software (i.e. Epoint) that creates a sort of ‘conversational marketing’ with consumers. Even if consumers are unable to find information from retailers, they can use the software to contact the company directly and in real-time via a chat.

Besides product and service innovation, some respondents particularly stressed the role of DT in enhancing the opportunities for product/service customization, as underlined in the tourism sector:

*The Travel Template software allows you to create very tailored solutions, by offering an editable technological infrastructure to the final customer.* (Eden Viaggi - Alpitour Spa)

Of course, the possibility to provide customized product innovation, aligned with the demand’s expectations, and the increasing ability to engage customers in the firm’s value creation process influenced firms-customers relationships, making them more stable, profitable, and beneficial to both.

Similar thoughts concern the communication policy. The use of digital tools for communication was widespread among the investigated companies. Eden Viaggi - Alpitour Spa reported devoting almost 70% of their communication expenditures on digital channels to date. According to the respondents, this trend has been strongly accelerated by the pandemic but it is likely to continue in the future, as digital technologies allow for quickly reaching the market worldwide with both standardised and targeted messages. Digital communication channels were perceived as effective for several reasons. First, they contribute to increase the brand visibility, giving companies the possibility to achieve new target markets and to align the brand identity with targets’ expectations:

*The online presence has allowed for improving the target market, especially reaching the youngest consumers.* (Doucal’s Srl)

*Digital technologies have allowed us to differentiate our branding strategy by cluster and community, which would have been difficult to achieve with traditional tools. Based on the above differentiation, we have developed different brands exploiting the potentiality of the digital presence.* (Go World)

In this way, companies improved brand-costumer interactions, enhancing the customer’s engagement with the brand as well as the frequency of interactions with the company.

Digital communication channels also enabled more interactive and personalised communication based on customer needs and interests. Notably, in Diasen Srl, the implementation of CRM software and the use of platforms such as Hubspot made it possible to develop ad hoc and geolocalised promotional activities and to obtain highly detailed information about prospects and customers. Moreover, digital tools facilitated the enrichment of interactive and multimedia content, including informative webinars about product offerings or the introduction of a virtual showroom:

*In times of pandemic, we had to adjust our communication strategy to always appear present and up to date in the eyes of customers. For this reason, we have developed new initiatives, such as the ‘Go World Emporium’ (an online shop with products from all over the world) and the ‘Go Europe’, which provides private flights aimed at high-spending targets, to show that we are active and there can be hope in the future, even if with different modalities from the classical tourism. In this period, moreover, there has been a lot of negative communication about travel; it was necessary to show that we can offer something new, especially enhancing local traditions and typical products of tourism destinations.* (Go World)

Despite some respondents reported using external agencies specialised in digital communication to fill internal gaps – as DT requires new skills for integrating online communication with the traditional one and properly employing the relative KPIs – companies realized that building long-term customer relationships is, today, unacceptable without the use of modern communication technologies and digital communication channels have become imperative among the firms analysed to provide customers with timely, personalized and meaningful information that increase trust, image, customers’ intimacy and commitment towards the company itself.

Firms-customers relationship improvement further benefited from the digitalization of sales activities. In our study, the area of sales was one of the most influenced by the DT process, which produced an increasing use of digital channels (e.g., e-commerce) besides the traditional ones, as well as the moving towards an omnichannel approach integrating online and offline channels in order to increase customer satisfaction and loyalty. It is worthy of notice the case of Salumificio Ciriaci Srl, which was recently exploring a potential collaboration with an international player, the Asian e-commerce giant Alibaba. While this collaboration has not materialised due to several regulatory issues, it suggests that firms – including small-sized ones – are really open to digitalisation. Alongside the integration of channels, DT has encouraged the improvement of new services related to sales processes (e.g. click & collect, click & home, click & drive), which has been further accelerated by the pandemic. Meanwhile, some firms (e.g., Biesse Spa) explicitly highlighted that DT also required an extensive revision of sales force management, implying the development of training programs to boost salespeople’s abilities and willingness to sell the digital solutions:

*Salespersons remained stuck in their product-oriented mindset. They considered machines selling more profitable and used digital services mainly to attract clients into buying extra products. The sales team showed a critical lack of motivation and did not comply with the new digital servitisation strategy until additional organisational measures (KPIs and training) were taken.* (Biesse Spa)

Anyway, digital technologies were critical to redesigning the information flow of data along the marketing channels. Firm-salespersons relationships have been reinforced, by means of more interactive communication and the adoption of sales-related CRM software – which improved reporting activities and the interactions between marketing, sales departments, and the overall sales control management – and the firm/customers relationships were generally reinforced since digital channels and services allow companies to generate customer value by designing more customised solutions and distribution plans.

#### Competencies and culture required to implement DT effectively

Notwithstanding the increasing adoption of digital technologies and the overall awareness of the respondents about the opportunities to use them for marketing purposes and customer relationship management, several concerns and organizational issues still limit their current use and exploitation. For example, although firms recognized the potential of digital technologies to improve market knowledge, in certain companies (e.g. Ariston Thermo Spa, Salumificio Ciriaci Srl), the use of digital tools to this end has been only recently implemented and needs to be further strengthened. In some cases (e.g. Go World), digital tools such as social media were considered unreliable and ineffective, especially when operating with niche targets, while other interviewed have not yet felt the need to use digital technologies for market analysis since they operate in traditional and mature sectors, such as the construction industry.

Some concerns also emerged in relation to branding management in the digital context, since managers feared the potential risk of damaging their brand identity if it is not consistent with the specific nature of each digital channel and related target market. Moreover, they were worried about the need to manage several touchpoints in an integrated way. Indeed, such integration efforts could require substantial human and time resources, which would result in high costs and long-term investments.

Sometimes, DT has been discussed in terms of potential threat also for business-customer relationships. For instance, customers who are reluctant or unable to adopt digital solutions may perceive digitalisation as an obstacle (Biesse Spa). In the tourism sector, disintermediation between agents and customers could worsen the customer experience:

*The journey begins when you buy, with the customers’ storytelling, which helps to choose and define the contents of the travel experience. The more one digitises, the more it becomes mass-produced and the more experience is lost, which is the heart of travel.* (Go World)

Similarly, in the retailing context, a company interviewed – that was traditionally founded on offline channels – warned of the risk of being unable to grasp the full potential of digital technology by failing to offer customers a usable digital service (e.g. e-commerce, click & pay). It declared to prefer personal dynamics of human relationships and relegated technologies to the role of simplifying processes, such as those of contract acquisitions and the monitoring of sales activities (Magazzini Gabrielli).

Some constraints to DT implementation have been also related to product specificities, as emerged from the analysis of Diasen Srl. It observed how DT has changed the process of introducing customers to the product. In particular, in the first phases of the funnel, some professionals (i.e. architects and engineers) who are more digitally advanced than construction companies and applicators have become ‘influencers’ and gained more power; however, closing negotiations and taking charge of the order still follow traditional logic and channels, as the products are generally extremely complex from a technical point of view.

However, what emerged from this study was a general gap in human skills and firms’ culture, which limits the use of digital tools and the effective implementation of the DT process. The analysed companies are partially aware of it and are trying to find potential solutions.

In terms of human resources, the companies believed they had a gap in digitisation and were taking action to fill them, regardless of size and industry specificities. They considered human resources as a critical means within DT processes, hence they are investing in training programs, which are generally outsourced to external partners, such as consultancy agencies, universities, and specialised research centers. In this respect, traditional figures such as Product Specialist has been transformed into Product Marketing Specialist by learning commercial and digital skills alongside technical ones. Some companies also resorted to external recruitment of already trained figures to occupy certain top-level positions in the organisation, in which they would act as ‘guides’ and ‘incentivise’ change, even for the remaining internal staff:

*For a change that affects the approach to work, and consequently the corporate culture, the seed must be grafted with already formed figures who bring that ‘spark’ also from the outside.* (Ariston Thermo Spa)

As an example, Diasen Srl introduced the Digital Marketing Specialist, while Biesse Spa established the Digital Marketing Manager (having the role of connector between sales, services, IT, and marketing) and the Customer Journey Builder (who had the task of creating automated e-mail paths to inform customers about the status of the contracts purchased).

However, the best option was a mix of internal training and the acquisition of new resources. Indeed, competencies are new, and the required profiles for managing DT are very complex:

*The knowledge and skills required concern the technological field to guide the progress of the platform, the business environment to obtain, analyse and interpret Big Data, and the organisational skills of control and management of change.* (Biesse Spa)

*There is certainly a basic skill, which is IT, that does not change, but digital is not just IT, on the contrary.* (Ariston Thermo Spa)

Therefore, both internal and external figures have been exposed to multidisciplinary training, which usually begins with an internal assessment of the organisation’s digital skills to both define a starting point and plan a series of training programmes that will be useful to lead the whole organisation to the next step.

Overall, the development of skills and capabilities integrating technological knowledge with the user experience, user interface, product, and service management implied the adoption of new organizational models for the marketing function as well as for the whole company, which shows increasing simplicity and openness of both internal and external borders. So, DT did not concern only the managerial sphere but also involved the whole organization and the training or acquisition of new resources has been closely linked to strategic planning.

Changes in human skills and structural models have been accompanied by process advances concerning the development of new ways to manage work in project teams as well as a widespread tendency (which, in some cases, is still a necessity) to review the cultural approach of the organisation to overcome the ‘culture of silos’ in favour of more organisational openness inspired by a data-driven orientation. As an expression of this cultural change, DT projects are often managed through a hybrid approach, where top-down leadership drives major changes, and bottom-up initiatives help to iron out the difficulties. This requires specific initiatives, as occurred in Biesse Spa, for instance, where a bi-weekly meeting between managers and employees was established to check on the progress of the DT roadmap. Such meetings allowed managers and employees to share their progress, doubts, and challenges and to effectively collaborate to solve problems.

#### The role of marketing in DT processes

The marketing function has been implemented in different ways within the companies. In some cases, it covered operational roles mainly related to communication and sales management (e.g., GoWorld); in others, it was a key-strategic function with advanced marketing intelligence and analytics tasks (e.g. Simonelli Group Spa). The increasing use of digital technologies usually changed the role and importance of marketing. Internal interactions with other departments have been substantially improved, thus enhancing the positive contamination of skills and knowledge:

*Marketing activities are traditionally widespread within Biesse, as it is a B2B company. The marketing department has been developed over the last years from a specific function that mainly dealt with communication activities (e.g. fair management) to a more integrated and integrative department, coordinating sales, service, and IT processes.* (Biesse Spa)

*The digitalization realized also internally by the company has facilitated the relationships and developed a sort of “business to employee” that has favoured the interchange and the development of knowledge between marketing and the other departments.* (Algam EKO Srl)

As a result, marketing operators have acquired a richer knowledge, integrating traditional skills and competencies (e.g. communication skills) with different ones, concerning the use of IT, the production process, as well as sales management.

*The marketing department has been developed over the last years from a specific function that mainly dealt with communication activities (e.g. fair management) to a more integrated and integrative department, coordinating sales, service, and IT processes.* (Biesse Spa)

The continuous interaction with IT, service and sales functions, and other departments – based on both internal workgroups and the employment of new professional figures with a role of connector – demonstrated essential to develop marketing strategies more in line with the digital innovation process. As a consequence, the role of marketing has improved and become strategically relevant for the companies’ success, since it helps companies to better orient their efforts towards the demand and to provide value propositions aligned with customers’ needs and expectations:

*Marketing coordinates the different activities both within the company and within the department itself. It performs communication activities and market analysis controls channels and sales management and plays as a supporter in the development/implementation of new projects. There is a central office that is organized in different subareas, based on their activity, but they usually work in teams, so there is a continuous and transversal exchange, and especially in the last 2 years the logic of connecting all touch points has been applied.* (Simonelli Group Spa)

*The marketing assistant allows us to strengthen the overall company’s market orientation and digital culture, generating quicker and more effective responses to the market*. (IT Consult)

Moreover, in some cases, marketing activities have become the catalyst of innovation, as they enhanced the overall understanding of customers, competitors, and market trends, which helps the management to define new products and service solutions. As the respondent of Magazzini Gabrielli stated:

*Over the last two years, marketing has assumed an important role in service and driving innovation.* (Magazzini Gabrielli)

Similarly, in Biesse Spa, the marketing department has been increasingly working as an enabler of innovative processes as it facilitates their spread and understanding both internally and towards customers. It actively responds to the firm’s need of creating a solid brand image and structure and to co-create value with customers.

Of course, some companies are still in the early stage of marketing development, as they operate in very traditional markets (e.g. Salumificio Ciriaci Srl) and, sometimes, they continue to confuse marketing with few operational activities such as communication (e.g. Doucal’s Srl, Diasen Srl). However, under the pressure of DT, all companies have recently increased their investment in marketing and it has been gradually involved in the strategic decision-making processes:

*The company is approaching marketing in recent years, following the generational change and the introduction of digital technologies. Our logo has been redesigned according to the logic of brand repositioning. The site has been entirely remade, including an emotional video able to attract more customers. We have opened a social account intending to communicate the quality of our products and the basic values that characterize us.* (Salumificio Ciriaci Srl)

Despite companies often entrusted to external agencies and expertises, as they have not yet developed the required marketing skills internally, DT helped them to acquire a solid awareness of the importance of marketing and of what this function can do for the long-term competitiveness of the company.

## Discussion and implications

### Theoretical implications

Our findings provide interesting and novel insights addressing the basic research questions underlying this study.

Regarding RQ1 (*How do firms conceive of DT? In particular, how and why they leverage digital technologies for it?*), our results confirm existing evidence that companies are facing heterogeneous paths of DT under the pressure of both exogenous and endogenous factors. They use a variety of digital technologies, some of which (e.g. AI, Big Data, blockchain systems, IoT) are still under-employed. In line with prior research (Gong & Ribiere, 2021; Verhoef et al., 2021), this finding suggests that DT is perceived and implemented by companies in different ways, but it is an ongoing process usually starting with digitisation and then arriving at the DT stage by way of the ‘digitalisation’ phase. The investigated companies that were using common technologies, such as social media and mobile and smart applications, were still in the intermediate step of ‘digitalisation’, which implies the encoding of analogic information into a digital format to improve existing business processes (Verhoef et al., 2021). Only few companies (e.g. Biesse and Simonelli Group) had nearly reached the ultimate stage of DT, which involves a strong adaptation of their internal procedures, organisation, and business model. Consistent with Hinterhuber et al., (2021), the interviewed companies are usually aware of the opportunities linked to digital technologies in terms of business process efficiency, professional ability improvements, and increases in the speed and control of processes. Yet, at the practical level, DT is not without pitfalls, as it implies important cultural and organisational changes. Moreover, managers tend to adopt a prudent attitude towards DT, as they recognise potential risks (e.g. loss of contact with customers, a potential digital divide within the company) that could negatively affect the long-term survival and competitiveness of the firm. The extant literature has mainly focused on motivators and triggering factors underlying DT. Thus, an original and novel contribution of the present study is its identification of such barriers to DT and the ways in which companies are trying to overcome them. As discussed later in this section, this contribution can inform practical suggestions and potentially useful interventions for companies undergoing a digitalisation process.

However, the most important contribution of this study is its overall investigation of the relationship between marketing and the DT phenomenon. Krishen et al., (2021) have recently noted that research on digital marketing is still growing, and more of these analyses are needed, as their findings have often been discordant. Moreover, the existing literature is quite fragmented, as studies have focused on single marketing activities or decisions, such as pricing (e.g. Abrate et al., 2012) or distribution (e.g. Hansen & Sia, 2015), rather than on marketing as a whole process and approach.

By contrast, this study investigated how digital technologies can reshape strategic and operational marketing activities, thus changing (i) the way firms/customers relationships are managed, (ii) how marketing skills and activities are organized, and (iii) the overall role and importance of marketing within the company.

As for the first issue (i.e. *RQ2: what are the impacts that DT is currently having on marketing activities and firm-customer relationships?)*, digital technologies offered companies an increasing amount of data and improved the ability to collect, store, process, and transmit information (Sheth, 2021; Wedel & Kannan, 2016). Such information can be easily conveyed and used to align offerings to a variety of demands by enhancing the coordination and synchronisation of production and distribution processes (i.e. the transfer from warehouses to distributors, order management, and related services). Overall, our results reveal that DT can improve a company’s responsiveness to customer needs, by promotingand new ways of managing the strategic and operational aspects of marketing processes. At the product level, for instance, the interviewed companies recognised that DT not only boosted simplicity in how customers learn about, find, purchase, and consume products and services but also increased efficacy in how products are conceived, realised, and tested. An example that was mentioned was the application of virtual reality to test products, which saved money and reduced lead time. At the communication level, digital channels received large attention by the investigated companies as they facilitate interactive and personalised communication. In line with prior studies (e.g., Zhang & Lin 2015) social media channels, for example, are currently employed by all the analysed firms, regardless of sector and firm-size, as they recognise the opportunity to allow customers to easily access to both products and information, which became particularly critical in times of pandemic crisis. Digital transformation also impacted the content of marketing messages, which nurtured brand strategies by focusing more on company values than on the technical features of products. At the same time, there was an awareness and concern regarding the need to manage many touchpoints in an integrated way. Concerning distribution, companies highlighted how DT encouraged an omnichannel approach to increasing customer satisfaction and loyalty. Furthermore, some companies observed that DT prompted an extension revision of sales management by uncovering the salient need for training programmes and new managerial positions. Finally, at the pricing level, DT enabled personalised offers and dynamic pricing. All this resulted in a more customized approach to the market, based on ongoing interaction with customers who can express their needs and participate in the value-creation process more easily and effectively than it was in the past. Besides that, the increasing amount of information can also impact the demand side by boosting consumer empowerment (Auh et al., 2019; Akhavannasab et al., 2018). The customer journey has been changed in both B2B and B2C contexts. Some of the bigger companies analysed talked about a ‘hybridisation’ of the customer journey because even in the B2B market, the huge availability of information pushes players to adopt typical paradigms of B2C environments. This hybridization imposes the organisation of marketing processes in a customer-centric way (Shah et al., 2006), even if it requires important investments. Notably, through CRM, customer knowledge can be constantly updated and diffused within a company, and marketing actions can be better targeted – often in real time. In this respect, CRM can be considered a kind of ‘facilitator’ to manage relationships and interact with customers at different stages of the customer journey (Nasir, 2015). Overall, in a DT context, the firm-customer relationships are usually strengthened, experiencing more stability and long-term satisfaction, as individual gratification is enhanced when people feel listened to (Collins, 2022).

The growing adoption of digital technologies also influenced how the marketing function is organised and its internal resources and capabilities are managed, providing empirical evidence answering our RQ3 (*What is impact of DT on marketing organization and competences?*). Enhancement of the strategic and analytical dimension of marketing tends to imply the need to improve cross-functional coordination between business units, which favours the adoption of informal integration mechanisms and a significant strengthening of coordination activities.

Consistent with Graesch et al., (2021), our findings reflect the importance of integrating marketing and IT areas to maintain a fair balance between digitisation and market objectives. If excessive weight is attributed to the technological dimension, there may be a risk of creating a value proposition that does not correspond to real customer needs. Hence, it is vital to improve communication between marketing and other corporate functions, especially those managing the digitisation process, to promote inter-functional coordination and the sharing of common objectives. This effort can sometimes require the introduction of new professional figures, the adaptation of existing skills through repeated training courses, or the development of a new approach to organisational problems based on overcoming ‘silo culture’ and opening up to flatter, flexible structures such that information can flow rapidly across the borders between marketing and other company departments. Over and above, to effectively realise DT, our study suggests an immense need for cultural change, as some scholars have remarked (Leeflang et al., 2014; Wedel & Kannan, 2016). In some cases among the analysed companies, the cultural inadequacy of management – mainly intended as a lack of culture of change that is necessary to reduce.

individual resistance to technology and related organizational changes – as proved to be a factor hindering the company’s ability to progress in the field of digital innovations. This finding underlines the need to start a process of acculturation for the internal staff of a company and the marketing area to promote the spread of a common culture, language, and way of thinking in compliance with the company’s innovative objectives.

Finally, the research findings recommend an overall improvement of the strategic role of marketing within the analysed companies, which provides evidence to address our RQ4 (*How might DT have changed the overall role and importance of marketing within firms?*). The interviews reflected a growing awareness of the strategic importance of marketing as a result of the recent pandemic crisis as well, which has made more evident the need to defend the competitive position with new and refined tools and methods (Savelli et al., 2021). However, under the pressure of DT, it further emerged that marketing no longer has the traditional role of aligning the variety allowed by technologies with consumers’ needs. Indeed, digital technologies are definitively replacing standardisation with customisation, thus improving opportunities to satisfy customers’ needs and wants. Hence, to some extent, digitalisation moderates the role of marketing as a ‘reducer’ of market complexity that increases the possibility of matching the production variance to diversified needs. Consequently, the role of marketing is changing to that of a creator of appropriate languages that allow for an effective interaction both between business functions and between firms and customers. Our findings suggest that marketing is increasingly becoming a connector of different skills and a strategic function for companies in strengthening the digital culture without losing sight of the market orientation and consumer culture (Shah & Murthi, 2021). When shifting the focus from the product to the language, the examined companies demonstrated a need to devote more attention to strategic marketing decisions (i.e. targeting and positioning) as well as achieve greater integration between marketing and other business units to increase collaboration and internal coordination. This perspective could imply that traditional marketing is in decline, which would leave space for a more interactive and less centralised approach in which customers (in both B2B and B2C settings) play an increasingly active role in the value creation process.

### Practical implications

Potential barriers and risks associated with DT can delay digital evolution and sometimes limit its exploitation, especially among the smallest firms. Therefore, there is a need for proper interventions at both the firm level and the public/institutional level. Specifically, the present findings indicate the urgency of three paths of intervention occurring at the cultural, organisational, and relational levels.

Overcoming existing cultural barriers and employee resistance to collaboration in DT is the basic condition. It might be achieved through the use of initiatives, such as internal communication campaigns that reinforce employees’ awareness of digital technologies or ad hoc rewards that encourage the commitment and interest of personnel in digital innovation. Senior and top-level management should assume a key role. By endorsing cross-functional projects, they could enhance inter-function collaborations and knowledge sharing, thus fostering cultural empathy.

Besides the cultural domain, organisational changes are also necessary to properly exploit DT opportunities. Such changes are critical since they involve both structural and human dimensions. The interviewed companies were suffering from a lack of competencies and technological skills. Moreover, a few cases highlighted the matter of the managerial approach to DT, from which different suggestions derive. In this respect, internal training activities should be improved to raise engagement and understanding of digital technologies and enhance the ability and willingness of employees to properly use digital solutions. Companies should offer internal training courses and promote external training activities by private and public institutions (e.g. higher education, universities). Since the introduction of new expertise demands economic efforts and long-term investments, activating collaboration with an external supplier while building internal competencies and know-how was a valid solution among the analysed companies. As for the managerial approach, the companies varied from top-down to bottom-up approaches. Maybe there is no one solution that is best for every situation. However, the same managers recommended a mixed-leadership approach as an effective solution. Such approach combines a bottom-up orientation with a top-down one; in this way, top managers can maintain a strategic role in guiding the DT process and minimising resistance, while bottom-up initiatives can facilitate engagement and commitment. To this end, the use of regular measurements (e.g. reports, meetings) might be useful for making incremental DT progress visible.

Finally, because the successful realisation of DT depends on an increasing number of actors, all subjects must collaborate towards the same goal and act as partners in the same project. This partnership requires a positive attitude towards co-creation, the ability to work together, and, above all, a stable and trust-based relationship network that assists firms in accessing digital resources. Particularly, at the inter-organisational level, communication with dealers, suppliers, and final customers could be augmented by regular visits, rewards programs, and training initiatives aimed at encouraging collaboration and long-term relationships.

## Conclusions, limitations, and future research directions

This study has investigated the complex relationship between DT and marketing through a multiple-case study approach. In summary, the contribution of this exploratory analysis is twofold. First, unlike existing studies, which have been fragmented and focused specifically on single activities or decisions, the present research considers marketing as a whole strategy and process, thus generating a holistic view of DT implications. The findings reveal some important changes concerning the role and organisation of marketing and the management of its activities. As part of a technologically intensive context in which both firm and customer behaviours are rapidly evolving, marketing should support the development of new business models and refine the traditional ones. Although marketing was implemented in varying ways among the investigated firms, DT enhanced its strategic role: marketing goals appeared less related to policy (i.e. 4P) management, the time horizon of decision-making became wider, analytical and control activities were carried out more systematically, and channel relationship management intensified. Moreover, organisational structures tended to flatten and be simplified, and the connections between marketing and other business functions become even more critical, which gestures to a systemic and customer-oriented approach as the most effective option. Overall, DT implies an improvement in the ability of marketing to more deeply and creatively understand the challenging trends of the society and the market. This change can enable a company to offer innovative products and services that meet the needs of the target demand, thus providing benefits to both customers and the company itself.

Second, the study has identified potential risks and barriers to DT encountered by the firms, especially the smallest ones. From these risks and barriers, we derive some useful recommendations for managers and policymakers. We particularly underline the need to intervene in three main dimensions: the cultural dimension to foster employees’ awareness and interest in digital technologies and inter-functional collaboration; the organisational dimension to fill gaps in competencies and technological skills and improve the managerial approach to DT, and the relational dimension to encourage collaboration and long-term relationships among the network of actors involved in DT.

Like any research, this study is subject to limitations, which can reveal fruitful opportunities for future research. First, the multiple case study research method presents some shortcomings. A main concern is the generalisation of results; while beyond the scope of our analysis, future research could seek to empirically validate the results with a larger sample. Moreover, this study considered 11 case studies within a single region, Marche, of a single country, Italy. Future studies could use a wider sample representing different geographical areas. Finally, it could be interesting for a future study to follow the transformation of investigated firms according to a longitudinal approach. Such research could illustrate the effects of the transformation in the medium to long term, including in terms of economic and competitive performance.

In conclusion, the findings of our research show that DT marks a new era of marketing evolution. The digital technological leap corresponds with increasing compliance between marketing theory and practice, especially among SMEs. It is well known that the predominance of SMEs has been a determining factor in the ‘*Marketing-non marketing all’italiana*’ (Varaldo et al., 2006), which features peculiar characteristics compared to the standard of managerial theory. Digital transformation seems to favour a realignment of the theoretical/conceptual and practical dimensions of marketing, which makes it easier and more practicable to conduct certain strategic activities, such as market analysis, market segmentation, and marketing mix customisation. This evolution has also highlighted the relevance of marketing for a firm’s competitiveness and success.
